# Identifying cancer driver genes based on multi-view heterogeneous graph convolutional network and self-attention mechanism

**DOI:** 10.1186/s12859-023-05140-3

**Published:** 2023-01-13

**Authors:** Wei Peng, Rong Wu, Wei Dai, Ning Yu

**Affiliations:** 1 Faculty of Information Engineering and Automation, Kunming University of Science and Technology, Kunming, 650050 China; 2 Computer Technology Application Key Lab of Yunnan Province, Kunming University of Science and Technology, Kunming, 650050 China; 3 Department of Computing Sciences, The College at Brockport, State University of New York, 350 New Campus Drive, Brockport, NY 14422 USA

**Keywords:** Cancer driver genes, Multi-view heterogeneous network embedding, Heterogeneous graph convolutional neural networks, Feature fusion, Network integration

## Abstract

**Background:**

Correctly identifying the driver genes that promote cell growth can significantly assist drug design, cancer diagnosis and treatment. The recent large-scale cancer genomics projects have revealed multi-omics data from thousands of cancer patients, which requires to design effective models to unlock the hidden knowledge within the valuable data and discover cancer drivers contributing to tumorigenesis.

**Results:**

In this work, we propose a graph convolution network-based method called MRNGCN that integrates multiple gene relationship networks to identify cancer driver genes. First, we constructed three gene relationship networks, including the gene–gene, gene–outlying gene and gene–miRNA networks. Then, genes learnt feature presentations from the three networks through three sharing-parameter heterogeneous graph convolution network (HGCN) models with the self-attention mechanism. After that, these gene features pass a convolution layer to generate fused features. Finally, we utilized the fused features and the original feature to optimize the model by minimizing the node and link prediction losses. Meanwhile, we combined the fused features, the original features and the three features learned from every network through a logistic regression model to predict cancer driver genes.

**Conclusions:**

We applied the MRNGCN to predict pan-cancer and cancer type-specific driver genes. Experimental results show that our model performs well in terms of the area under the ROC curve (AUC) and the area under the precision–recall curve (AUPRC) compared to state-of-the-art methods. Ablation experimental results show that our model successfully improved the cancer driver identification by integrating multiple gene relationship networks.

**Supplementary Information:**

The online version contains supplementary material available at 10.1186/s12859-023-05140-3.

## Background

It is generally accepted that cancer arises due to the accumulation of genetic mutations [1]. However, not all mutations cause cancer to develop. Mutations essential for cancer development are known as driver mutations, and genes with driver mutations are known as driver genes. Correctly identifying the cancer driver genes can assist drug design, cancer diagnosis and treatment. The recent large-scale cancer genomics projects, such as The Cancer Genome Atlas (TCGA) [2], the International Cancer Genome Consortium (ICGC) [3] and the Catalogue Of Somatic Mutations In Cancer (COSMIC) [4] have revealed multi-omics data from thousands of cancer patients. The multi-omics data, such as genomic, transcriptomic and proteomic data, has been widely applied by computational methods to identify driver genes.

The early stage methods identify cancer drivers assuming that cancer drivers frequently undergo genomic alterations across many samples. These methods, such as MuSiC [5] or ActiveDriver [6], identify cancer drivers by measuring the difference between the gene mutations compared to their predefined background mutation rates. However, the frequency-based methods often fail to correctly estimate background mutation rates and ignore the cancer drivers with low mutation frequencies. Other methods like HotNet2 [7] and MUFFINN [8] assume that cancer driver genes are often enriched in protein complexes or pathways. They project the mutated genes onto a protein–protein interaction (PPI) network and detect driver genes by finding the highly significant mutated gene modules or essential genes that strongly influence other genes. However, these network-based methods are limited by the reliability of the PPI network. To improve PPI reliability and to fully use the valuable multi-omics data, some methods use multi-omics data to weight PPI and filter out the noisy connections under the constraint of co-expression, co-subcellular and co-tissue [9, 10] among the molecules. However, these methods do not efficiently exploit the relationships between multi-omics data to boost the accuracy of the driver gene prediction.

The recent network representation techniques become popular for learning low-dimensional vectors for the network nodes. This technique can screen network noise and has been successfully applied to detect cancer drivers. RLAG [11] runs node2vec [12] on the attribute network and PPI network simultaneously to learn gene feature representations and predict the cancer driver genes. DeepDriver [13] concatenates the features of the gene and its k nearest co-expression neighbours to generate a feature matrix for every gene. Then, it adopts a convolution neural network model to learn gene features for driver gene prediction. The emerging Graph Convolutional Networks (GCN) models [14], i.e., EMOGI [15] and MTGCN [16], can learn features of network nodes by naturally combining the network structure with node features and achieve good performance in cancer driver prediction. Nevertheless, these GCN-based methods only aggregate features from the homogeneous neighbours in the gene–gene network. Tumorigenesis usually involves complex interactions between genes and other molecules. For example, the cancer driver genes cause dysregulation of their downstream gene expression. MiRNAs regulate the expression of their target genes, and the dysfunctions of genes can trigger cancers. Hence, it is crucial to employ multi-omics data to construct multiple heterogeneous networks describing the relationships between genes and other molecules and design effective models to integrate these networks to discover cancer drivers. Early methods construct multiple homogeneous networks from multi-omics data and integrate the networks to classify cancer subtypes [17, 18]. Some multi-relational GCN-based and random walk-based methods predict drug targets and miRNA-disease associations from the heterogeneous networks [19]. However, few approaches integrate multiple heterogeneous networks to predict cancer driver genes. With the advancement of deep learning in various tasks, some methods leverage the power of multi-layer neural networks for multi-omics data integration and feature learning. However, few exploit the correlations between different omics data.

Hence, we proposed a novel method to predict cancer driver genes based on multiple gene relationship networks and heterogeneous graph convolution models (MRNGCN). We first constructed three gene relationship networks: a gene–gene network, a gene–outlying gene network and a gene–miRNA network. These networks describe gene features related to cancer development and progression from different views. Then, genes learn feature presentations from the three networks through three sharing-parameter HGCN models with the self-attention mechanism. After that, the three gene features pass a 2D-convolution layer to generate fused features. We leverage the fused and original features to optimize the model. Finally, a logistic regression (LR) model combines the fused features, the original features and the three features learned from every network for cancer driver genes prediction. To our knowledge, MRNGCN is the first algorithm that integrates the relationships between gene and gene, gene and outlying gene, gene and miRNAs to predict cancer drivers. Compared with previous methods, our main contributions are summarized as follows:

1. Besides the gene–gene network, we introduced the gene–outlying network and gene–miRNA network to identify cancer driver genes. These networks describe gene features related to cancer development and progression from different views. Moreover, we prepared multi-omics data features for the genes, outlying genes and miRNAs in the three networks, considering the corresponding biological characteristics.
2. We proposed a novel method to predict cancer drivers by integrating three gene-related networks based on the heterogeneous graph convolution network (HGCN) model and the self-attention mechanism. These HGCN models sharing parameters can extract common features of the three networks, and the self-attention mechanism can consider the relationships between network nodes with long distances.
3. We leveraged a logistic regression (LR) model to combine the fused features, original features and three features learned from networks to predict cancer driver genes. The coefficients in the LR model interpret every part’s contribution (see Additional file 1).
4. We conducted extensive experiments to test our model. The results show that our method performs better than state-of-the-art methods in cancer driver prediction on the pan-cancer, most cancer types and the independent datasets.

## Methods

**Fig. 1:**
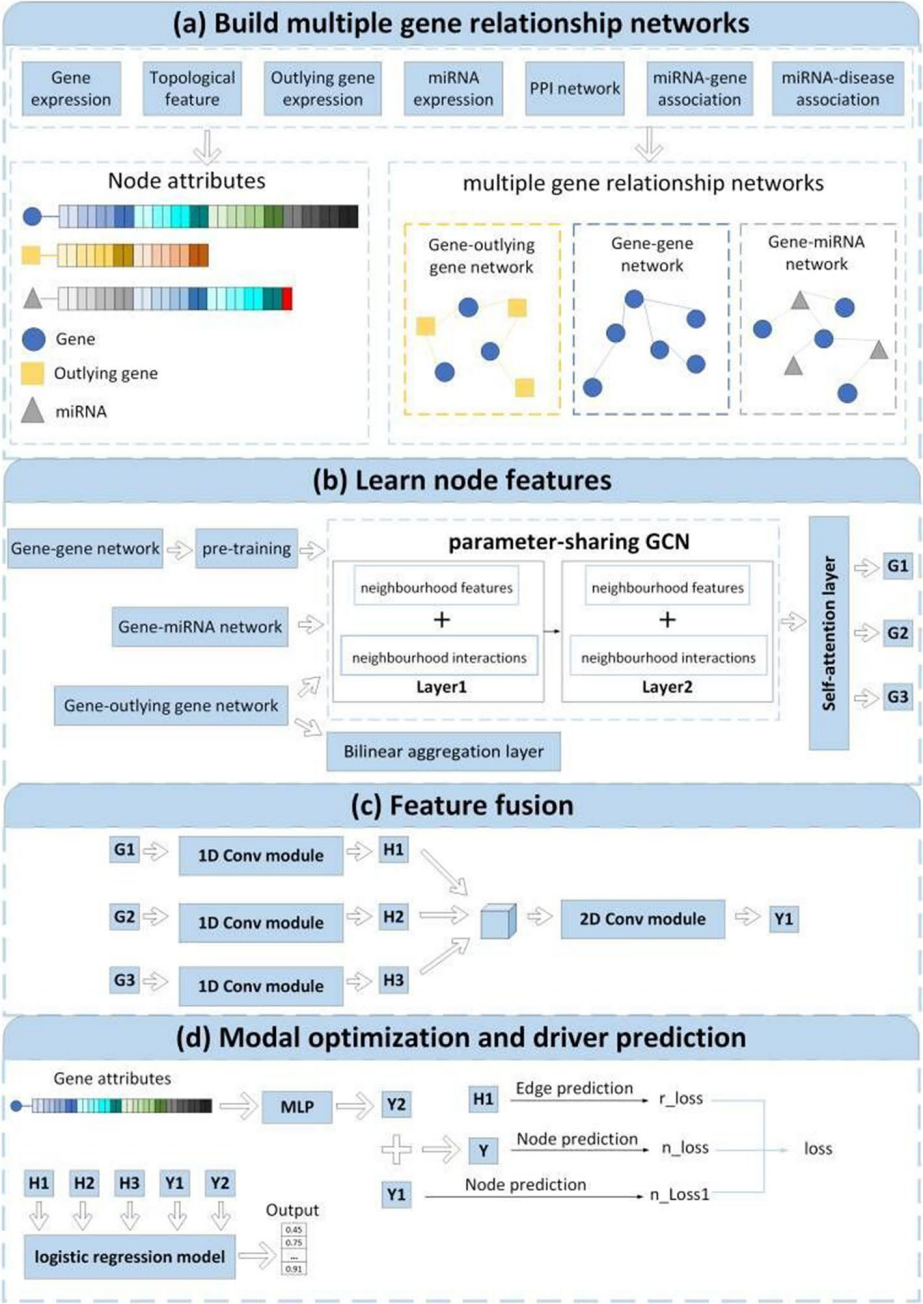
The framework of MRNGCN. Our model is divided into four parts: **a** constructing three relational networks and preparing node attributes, **b** learning the features of gene nodes in each network, **c** fusing the gene features learned fromthe three networks, and **d** optimize model parameters and predict cancer drivers

Figure 1 illustrates the framework of our approach MRNGCN. First, MRNGCN builds three gene relationship networks: a gene–gene network, a gene–outlying gene network and a gene–miRNA network. Then, it learns gene features from the three networks through three parameter-sharing heterogeneous graph convolution network models and a shared self-attention layer. Next, our model jointly utilizes 1D and 2D convolution operations to fuse the gene features learned from the three networks. Finally, we leverage the fused gene features, the original gene features and the gene features learned from the gene–gene network to optimize the model by minimizing the node and link prediction. Meanwhile, a logistic regression model combines these features to predict cancer driver genes.

### Experimental data

We downloaded gene mutation, DNA methylation data, and miRNA expression data from TCGA [2], containing more than 8,000 samples for 16 cancer types. Gene expression data were obtained from Wang et al. [20], which were collected from TCGA and were further normalized and batch corrected by ComBat [21]. Like MTGCN [16], we only focused on cancer types for which gene expression data and DNA methylation information are available in both tumor and normal tissues. Hence, this work involves 16 cancer types. We constructed the gene–gene network based on PPI data from the Consensus Path DB (CPDB) [22] database. We got 13,627 genes and 504,378 gene–gene edges with interaction scores above 0.5. We used the genes in the PPI networks for cancer driver prediction. The miRNA-gene associations from the mirTarbase V8.0 database [23] contain 2,599 miRNAs, 15,064 genes, and 380,639 miRNA-gene associations. The miRNA-disease associations were from the HMDD database version 3.0 (http://www.cuilab.cn/hmdd). The benchmark driver gene of pan-cancer was from MTGCN [16] Additional file 1. They were 796 high confidence driver genes in NGC 6.0. To obtain a negative sample list, we started with all genes and recursively removed genes from the NCG, COSMIC, OMIM databases and KEGG cancer pathways. Hence, our pan-cancer dataset consists of 796 positive and 2,187 negative samples. Cancer type-specific positive samples were from NCG 6.0 tagged with that cancer type and shared the same negative samples with pan-cancer.

### Building multiple gene relationship networks

#### Gene–gene network

We used the PPI network to construct the gene–gene network. Let $A_{PP} \in \left\{ {0,1} \right\}^{n \times n}$ be the adjacency matrix of the gene–gene network with the number n of genes. If two genes connect through an edge in the PPI network, the corresponding value in the matrix $A_{PP}$ is 1. Otherwise, it is 0. We used MTGCN [16] to prepare initial attributes for gene nodes in the network (See Additional file 1 for details). Let $X_{P} \in R^{{n \times F_{1} }}$ denote the initial gene attributes consisting of biological and topological features. For each cancer type, we calculated gene mutation rate, differential DNA methylation rate, and gene differential expression rate as the biological features of the genes. Since we only focused on 16 cancer types, each gene had a 48-dimensional biological feature vector, including 16 mutation rates, 16 methylation values, and 16 differential expression rates, which were min–max normalized. The 16-dimensional topological features of genes resulted from the note2Vec on the gene–gene network. We concatenated the biological and topological features to get the 64-dimensional initial attributes of genes.

#### Gene–outlying gene network

We considered that a driver gene usually affects the expression of genes linked to it in a biological network, so we constructed a gene–outlying gene network. A gene is considered to be outlying if the absolute value of its z-score is above the threshold (this work sets the threshold to 2) [10]. We collected all outlying genes at least expressed abnormally in one sample of the 16 cancer types. Let $A_{PO} \in \left\{ {0,1} \right\}^{n \times m}$ be the adjacency matrix of the gene–outlying network with the number n of genes and m of outlying genes. We connected a gene and an outlying gene and set the corresponding value of $A_{PO}$ as 1 if the gene mutates in at least one cancer sample and links to the outlying gene in the PPI network. Hence, the gene–outlying gene network of pan-cancer contains 13,627 genes, 12,248 outlying genes and 469,078 edges. We initialized the attributes of the gene nodes in the gene–outlying network as $X_{P}$, the same attributes of the gene nodes in the gene–gene network. The initial attributes of the outlying genes consist of the average z-scores across all samples of a cancer type and the frequencies of being outlying among the samples of the cancer type. Let $X_{O} \in R^{{m \times F_{2} }}$ be the vector of the initial attributes of the outlying genes, which would consist of 16 z-score features and 16 frequency features in the pan-cancer dataset. For convenience, the outlying gene initial features underwent linearly transformation from 32 dimensions to 64, the dimension of initial gene features.

#### Gene–miRNA network

We constructed a gene–miRNA network considering the regulatory relationship of miRNAs on gene expression. The known associations between miRNAs and their targeted genes were from mirTarbase V8.0 [23]. Let $A_{PR} \in \left\{ {0,1} \right\}^{n \times t}$ be the adjacency matrix of the gene–miRNA network with n genes and t miRNAs. The genes were the mutated gene of TCGA samples. The miRNAs were those that both appeared in the mirTarbase database and the TCGA samples. Hence, the gene–miRNA network involves 1390 miRNAs and 153,913 edges connected with the 13,627 genes. The value of $A_{PR}$ is 1 if a gene is associated with a miRNA. Otherwise, it is 0. We also initialized the attributes of the gene nodes in the gene–miRNA network as $X_{P}$. The initial attributes of miRNAs denoted by $X_{R} \in R^{{t \times F_{3} }}$ include the average z-scores and the average different expression values across all samples of every cancer type and the similarities with other miRNAs. Since miRNAs link to the pathologies of cancers by regulating the expression of their targeted genes and the dysfunction of similar miRNAs would lead to a similar phenotype, we introduced the miRNA similarities as part of initial miRNA attributes. Similar to previous works [19], the miRNA similarity was measured by the miRNA GIP (Gaussian Interaction Profile) kernel based on known miRNA-disease associations. We linearly transform the miRNA GIP similarity matrix and obtained 16-dimensional miRNA similarity features to avoid bias. Finally, for the pan-cancer with 16 cancer types, $X_{R}$ would have 16 z-scores and 16 differential expression values and the miRNA similarities of length 16 and the number of genes connected to each miRNA. Similarly, for convenience, we linearly transformed the miRNA initial features to the same dimension as initial gene features. To fully use the valuable gene–miRNA associations, we input $X_{P}$ and $X_{R}$ into a two-layer heterogeneous graph convlutional network (HGCN) model the same as the model in section "the heterogeneous graph convolutional network" to implement pre-training on the gene–miRNA network and learn new features for genes and miRNA, called $X_{{P_{pre} }}$ and $X_{{R_{pre} }}$. Hence, we used $X_{{P_{pre} }}$ and $X_{{R_{pre} }}$ as the gene and miRNA initial features of the gene–miRNA network for following feature learning (See Additional file 1 for details).

### Learning node features from multiple networks

#### The heterogeneous graph convolutional network

We employed three two-layer heterogeneous graph convolutional network (HGCN) modules to learn feature representations for the nodes of the three relationship networks. The HGCN modules update the node features by aggregating both neighbourhood features and neighbourhood interactions. To extract the common features of the three relationship networks, we input the three networks and their initial node attributes into three parameter-sharing HGCN models.

Aggregating neighbourhood feature captures the interaction pattern of the node in the network. We started the aggregation by normalizing the adjacency matrix of the three networks. Let $A_{ij} \in \left\{ {A_{PP} ,A_{PO} ,A_{PR} } \right\}$ be one of the adjacent matrices of the three networks. $P_{ij} \in \left\{ {P_{PP} ,P_{PO} ,P_{PR} } \right\}$ is its normalized matrix. $P_{ij} = D_{i}^{{ - \frac{1}{2}}} A_{ij} D_{j}^{{ - \frac{1}{2}}}$, $D_{i} = \mathop \sum \limits_{j} A_{ij} + 1$ and $D_{j} = \mathop \sum \limits_{i} A_{ji} + 1$. Since $A_{ji} = A_{ij}^{T}$, then $P_{ji} = P_{ij}^{T}$. We took Eqs. (1)–(3) to aggregate neighbourhood features for the nodes in the gene–gene, gene–outlying gene and gene–miRNA networks, respectively. Here, $\theta_{k} \in R^{F1 \times F2}$ are shared by the three networks.

1 $$AGG_{NF_{P1}} = P_{PP} X_{P} \theta_{k}$$

2 $$AGG_{{NF_{P2} }} = P_{PO} X_{O} \theta_{k} ,\quad AGG_{{NF_{O} }} = P_{OP} X_{P} \theta_{k}$$

3 $$AGG_{{NF_{P3} }} = P_{PR} X_{{R_{pre} }} \theta_{k} , \quad AGG_{{NF_{R} }} = P_{RP} X_{{P_{pre} }} \theta_{k}$$

To further capture the interaction patterns of the network nodes, we considered the neighbourhood interactions, which were measured by the element-wise dot product between the node features and its neighbours' features. Equations (4)–(6) aggregate neighbourhood interactions for nodes of the gene–gene, gene–outlying and gene–miRNA networks, respectively. Here $W_{1} \in R^{F1 \times F2}$, $b_{1} \in R^{F1 \times F2}$ are shared by the three networks. $\odot$ denotes the element-wise product.

4 $$AGG_{{NI_{P1} }} = P_{PP} X_{P} \odot X_{P} W_{1} + b_{1}$$

5 $$AGG_{{NI_{P2} }} = P_{PO} X_{O} \odot X_{P} W_{1} + b_{1} ,\quad AGG_{{NI_{O} }} = P_{OP} X_{P} \odot X_{O} W_{1} + b_{1}$$

6 $$AGG_{{NI_{P3} }} = P_{PR} X_{{R_{pre} }} \odot X_{{P_{pre} }} W_{1} + b_{1} , \quad AGG_{{NI_{R} }} = P_{RP} X_{{P_{pre} }} \odot X_{{R_{pre} }} W_{1} + b_{1}$$

Finally, the HGCN models learned features for nodes of the three networks by aggregating neighbourhood features and interactions. Mathematically, the process can be defined as follows.

7 $$H\left( X \right)_{V} = HGCN_{V} (\{ H\left( X \right)_{i} \}_{i \in N\left( V \right)} ) = \sigma (AGG_{{NF_{V} }} + AGG_{{NI_{V} }} )$$

where $V \in \left\{ {P1,P2,P3,O,R} \right\}$ denotes the target node, $H\left( X \right)_{V}$ denotes the features of the node $V$ learned from the corresponding network through the HGCN model. $H\left( X \right)_{i}$ is features of the neighbores of the target node, and $N\left( V \right)$ denotes neighbor set of the node $V$ in one of the three networks, and $\sigma$ denotes the activation function, e.g. ReLU.

Our graph convolution module contains multiple graph convolution layers. Setting the number $L$ of graph convolution layers, $H\left( X \right)_{P1}$, $H\left( X \right)_{P2}$ and $H\left( X \right)_{P3}$ denote the final gene features learned by the HGCN models from the gene–gene, gene–outlying gene and gene–miRNA networks, respectively. Equation (8) express the process. Here, $L = 2$.

8 $$\left\{ {\begin{array}{*{20}l} {H\left( X \right)_{P1} = HGCN_{P}^{L} \left( {\left\{ {HGCN_{P}^{L - 1} \cdots HGCN_{P}^{1} \left( {\left\{ {X_{P} } \right\}_{P \in N\left( P \right)} } \right)} \right\}_{P \in N\left( P \right)} } \right)} \hfill \\ {H\left( X \right)_{P2} = HGCN_{P}^{L} \left( {\left\{ {HGCN_{P}^{L - 1} \cdots HGCN_{P}^{1} \left( {\left\{ {X_{O} } \right\}_{O \in N\left( P \right)} } \right)} \right\}_{O \in N\left( P \right)} } \right)} \hfill \\ {H\left( X \right)_{P3} = HGCN_{P}^{L} \left( {\left\{ {HGCN_{P}^{L - 1} \cdots HGCN_{P}^{1} \left( {\left\{ {X_{{R_{pre} }} } \right\}_{R \in N\left( P \right)} } \right)} \right\}_{R \in N\left( P \right)} } \right)} \hfill \\ \end{array} } \right.$$

#### Bilinear aggregation layer

Since the cancer driver genes cause abnormal expression of their downstream genes and lead to cancer development, we constructed the gene–outlying gene network to learn gene features that help detect cancer driver genes. The outlying genes working together contribute to cancer progression [9, 10]. To take advantage of the interactions between the outlying genes, we introduced a bilinear graph neural network (BGNN) [24] layer to learn feature representations for the nodes of the gene–outlying network. In Eq. (9), genes in the gene–outlying network can also aggregate the interaction features between their neighbors. Here, we considered the gene nodes themselves and merge them into the neighbor set to obtain the extended neighbourhood $\tilde{N}\left( P \right)$. Moreover, we ignored self-interactions in the neighbourhood to avoid introducing additional noise. The bilinear aggregation features of genes in the gene–outlying gene network defined as follows.

9 $$H\left( X \right)_{P}^{BA} = \frac{1}{{b_{P} }}\mathop \sum \limits_{{i \in \tilde{N}\left( P \right)}} \mathop \sum \limits_{{j \in \tilde{N}\left( P \right)\& i < j}} \left( {X\left( i \right)W + b} \right) \odot \left( {X\left( j \right)W + b} \right)$$

where $W$ and $b$ denote the learnable parameters, ⊙ is the product of elements. and are two different node indices from $\tilde{N}\left( P \right)$. $X \in \{ X_{P} ,X_{O} \}$ is the initial features of genes or outlying genes. $b_{P} = \frac{1}{2}\tilde{d}_{p} \left( {\tilde{d}_{p} - 1} \right)$ denotes the number of node interactions.

Hence, the genes in the gene–outlying gene network can learn two features from the network. The one is $H\left( X \right)_{P2}$, learned by a two-layer HGCN. The other is $H\left( X \right)_{P}^{BA}$, learned by a BGNN layer. We summarized the two features and balanced their weights using a parameter to get the final gene features, ${\tilde{\text{H}}}\left( X \right)_{P2}$, from the gene–outlying network.

10 $${\tilde{\text{H}}}\left( X \right)_{P2} = \left( {1 - \alpha } \right)*H\left( X \right)_{P2} + \alpha *H\left( X \right)_{P}^{BA}$$

#### Self-attention layer

After running HGCN models on the three relational networks, we learnt gene features $H\left( X \right)_{P1}$, ${\tilde{\text{H}}}\left( X \right)_{P2}$ and $H\left( X \right)_{P3}$ from the gene–gene, gene–outlying gene and gene–miRNA networks, respectively. Previous studies observed that driver genes often work together to form protein complexes or are enriched in some signal pathways. To use the interactions between genes and pay more attention to the crucial interactions when learning features for genes, we took the three features as inputs of a self-attention layer seperately. The self-attention module can naturally combine all gene features from a network as inputs, allowing the inputs to interact with each other and find out who they should pay more attention to. For example, we took the gene features from the gene–gene network as inputs of the self-attention model (See Eq. (11)). The self-attention model multiplies every input with $W^{Q}$, $W^{K}$ and $W^{V}$ to obtain its query($Q_{1}$), key($K_{1}$) and value($V_{1}$) representations. The genes in the gene–gene network(Attention($Q_{1}$, $K_{1}$, $V_{1}$) generated their contextual representations through multiplication between the weighted attention-score matrix and all inputs' values. The weighed attention-score matrix was calculated by applying a softmax on a dot product between the queries with all inputs’ keys, divided each by $\sqrt d$.

11 $$\left\{ {\begin{array}{*{20}l} {\left[ {Q_{i} ,K_{i} ,V_{i} } \right] = H\left( X \right)_{Pi} \left[ {W^{Q} ,W^{K} ,W^{V} } \right]} \hfill \\ {Attention\left( {Q_{i} ,K_{i} ,V_{i} } \right) = softmax(Q_{i} K_{i}^{T} /\sqrt d )V_{i} } \hfill \\ \end{array} } \right.$$

where i = 1,2,3, which represents self-attention layer for three networks, $d$ is the dimensionality of $Q$ and $K$. $K_{*}^{T}$ is the matrix transpose. To preserve the uniqueness of the gene features learned for every network, we added the gene features before the self-attention layer with the gene features after the self-attention to obtain the gene features $H\left( X \right)_{att}^{i} , i = 1,2,3$ for the gene–gene, gene–outlying gene and gene–miRNA networks, respectively (see Eq. (12)).

12 $$H\left( X \right)_{att}^{i} = Attention\left( {Q_{i} ,K_{i} ,V_{i} } \right) + H\left( X \right)_{Pi} .$$

#### Feature fusion

After passing the initial gene features through the HGCN model and self-attention layer, we obtained three gene features from the three networks, denoted by $H\left( X \right)_{att}^{1}$, $H\left( X \right)_{att}^{2}$, $H\left( X \right)_{att}^{3}$. Then, we employed three 1D-convolution modules to reduce the dimensions of the three gene features and a 2D-convolution module to fuse the gene features.

Every 1D-convolution module consists of two convolution layers. The size of the convolution kernel for both convolution layers is 1. The number of input channels of the module is the dimension of the feature matrix and the number of output channels is 1. The learned gene features are denoted as $H\left( X \right)_{1D}^{1}$, $H\left( X \right)_{1D}^{2}$, $H\left( X \right)_{1D}^{3}$ for the gene–gene, gene–outlying gene and gene–miRNA networks, respectively.

We integrated the three gene features from the output of the 1D-convolution module to generate fused gene features (denoted as $H\left( X \right)_{2D}$) through a 2D-convolution module. The 2D-convolution module consists of a two-dimensional convolutional layer (see Additional file 1: Figure S1). Firstly, we stack the three gene features from the output of the 1D-convolution module (i.e., $H\left( X \right)_{1D}^{1}$, $H\left( X \right)_{1D}^{2}$, $H\left( X \right)_{1D}^{3}$) to obtain a feature matrix $H\left( X \right)_{stack} \in R^{3 \times n \times 1}$, with $n$ denoting the number of gene nodes. Then we padded out a circle of zeros around the $H\left( X \right)_{stack}$ and implemented two-dimensional convolutional operations on the $H\left( X \right)_{stack}$. The convolution kernel size is $\left( {w_{c} \times h_{c} } \right)$ and the perceptual field of $H\left( X \right)_{stack}$ is $3 \times w_{c} \times h_{c}$. Here, we set $w_{c} = h_{c} = 3$. The input channel is 3, and the output channel is 1. We summed the feature maps on each channel to form the fused gene features, $H\left( X \right)_{2D} \in R^{n \times 1}$.

### Model optimization and driver gene prediction

#### Model optimization

After passing the initial input gene features through the HGCN modules, the self-attention layer and the feature fusion module, we obtained the gene feature representations containing the network context information ($H\left( X \right)_{2D}$). The original gene features characterizing the gene themselves also play an important role in driver gene identification. Hence we pass the initial gene features through a three-layer multi-layer perceptron (MLP) to get another gene feature representations, called $H\left( X \right)_{mlp}$, defined in Eq. (13).

13 $$H\left( X \right)_{mlp} = Linear^{3} \left( {\sigma \left( {Linear^{2} \left( {\sigma \left( {Linear^{1} (X_{P} )} \right)} \right)} \right)} \right)$$

where $X_{P}$ stores initial gene features. We added $H\left( X \right)_{mlp}$ with $H\left( X \right)_{2D}$ to generate synthesis gene feature representations denoted by $H\left( X \right)_{syn}$ and used the synthesis gene features to predict cancer driver genes after passing the sigmoid function. A binary cross-entropy was employed to quantify the node prediction loss.

14 $$L_{n\_loss} \left\{ {H\left( X \right)_{syn} } \right\} = - \frac{1}{n}\mathop \sum \limits_{i = 1}^{n} \left[ {y_{i} log\left( {\widehat{{y_{i} }}} \right) + \left( {1 - y_{i} } \right)log\left( {1 - \widehat{{y_{i} }}} \right)} \right]$$

where $\widehat{{y_{i} }}$ is the predicted score of the gene $i$, and $y_{i}$ is its real label whose value is 0 or 1, $n$ is the number of genes in the training dataset. To ensure the reliability of the gene features learned in the network context and their independent predictive power, we applied the sigmoid function on the $H\left( X \right)_{2D}$ for cancer gene predictions. The node prediction loss was calculated as follows.

15 $$L_{n\_loss1} \left\{ {H\left( X \right)_{2D} } \right\} = - \frac{1}{n}\mathop \sum \limits_{i = 1}^{n} \left[ {y_{i} log\left( {\widehat{{y_{i} }}} \right) + \left( {1 - y_{i} } \right)log\left( {1 - \widehat{{y_{i} }}} \right)} \right]$$

The HGCN model updates the representation of the gene nodes in the networks by aggregating their neighbors' features and interactions with their neighbors. However, this model does not ensure that the learned gene features maintain the original network structure. Moreover, the model parameters are optimized on the limited number of known driver gene labels. To solve this problem, we implemented an inner product between the gene features learned from the gene–gene network, (i.e. $H\left( X \right)_{1D}^{1}$) to predict the network links [16]. The reconstructed adjacency matrix is $\hat{A}_{PP}$.

16 $$\hat{A}_{PP} = \sigma (\{ H\left( X \right)_{1D}^{1} \} \{ H\left( X \right)_{1D}^{1} \;^{T} \} )$$

where $\sigma$ is the sigmoid function. We then calculated the binary cross-entropy loss of the link prediction (see Eq. 17)).

17 $$L_{r\_loss} \left\{ {H\left( X \right)_{1D}^{1} } \right\} = - \frac{1}{n}\left\{ {\mathop \sum \limits_{i,j \in E} \left\{ {log\hat{a}_{i,j} } \right\} + \mathop \sum \limits_{i,j \in Neg} \left\{ {1 - log\hat{a}_{i,j} } \right\}} \right\}$$

where $E$ is the edge set of the gene–gene network, and $n$ is the size of $E$. $Neg$ is the set of negative samples with size $n$, obtained by negative sampling, and $\hat{a}_{i,j}$ is the value of the reconstructed adjacency matrix.

Our final loss function consists of two node-prediction losses and a link-prediction loss, defined in Eq. (18). $\omega_{1}$ and $\omega_{2}$ are hyper-parameters, regulating the weight of each loss term in training.

18 $$L_{total} = L_{n\_loss} + \omega_{1} *L_{n\_loss1} + \omega_{2} *L_{r\_loss} .$$

#### Predicting cancer driver genes

Our MRNGCN approach is composed of several modules. Every modules encodes different kinds of gene features. These features character genes' roles in cell life from different views. For example, the gene features from the 1D-convolution modules (i.e. $H\left( X \right)_{1D}^{1}$, $H\left( X \right)_{1D}^{2}$, $H\left( X \right)_{1D}^{3}$) represent gene characteristics in the three networks. The 2D-convolution module produces the fused gene features $H\left( X \right)_{2D}$, and $H\left( X \right)_{mlp}$ represents the original gene features. We leveraged a logistic regression (LR) model to combine these gene features to predict cancer driver genes in the test set. The definition of the LR model is as follows.

$$x = w_{1} H\left( X \right)_{1D}^{1} + w_{2} H\left( X \right)_{1D}^{2} + w_{3} H\left( X \right)_{1D}^{3} + w_{4} H\left( X \right)_{2D} + w_{5} H\left( X \right)_{mlp} + \varepsilon$$

19 $$f\left( x \right) = \frac{1}{{1 + e^{ - x} }}$$

where ${w}_{1}$, ${w}_{2}$, ${w}_{3}$, $w_{4}$, $w_{5}$ are weights of the LR model, illustrating the contribution of each feature to the driver gene identification. See Additional file 1 for the pseudo-code of MRNGCN.

## Experiments

### Baselines

To evaluate the performance of our model, we compared it with MTGCN [16], EMOGI [15], GCN [25], GAT [25], RGCN [25], Multi-omics fusion and MOGONET [17]. MTGCN and EMOGI are the most advanced GCN-based methods for cancer driver predictions. GCN and GAT are two typical GCN models. MTGCN, EMOGI, GCN and GAT all run on the gene–gene network. RGCN integrates the gene–gene, gene–outlying gene and gene–miRNA networks into a heterogeneous network. It runs a relational GCN model on the network and assigns suitable weights to different types of relationships when aggregating neighbor features. MOGONET was originally proposed to integrate multi-omics data for cancer subtype classification. To evaluate the effectiveness of our model, we input the gene features learned from the three networks by our model into the feature fusion module of MOGONET to predict cancer driver genes (See Additional file 1: Figure S3). Multi-omics fusion concatenates the gene features learned from the three networks (i.e., $H\left( X \right)_{att}^{1}$, $H\left( X \right)_{att}^{2}$, $H\left( X \right)_{att}^{3}$) and the original gene features, $X_{P}$ and then passes them through three fully connected layers with 256, 64, and 1 units to acquire gene fused features. It utilizes the fused features and $H\left( X \right)_{att}^{1}$ to minimizing the node prediction loss and the link predict loss, respectively. (See Additional file 1: Figure S2).

### Parameters setting

Our model was implemented based on the PyTorch framework. The optimizer for our model was Adam. The optimal combination of hyper-parameters was as follows: the dropout rate was set to 0.5 by default, except for the self-attention layer, which was set to 0.2. Our model, multi-omics fusion and MOGONET all had two graph convolution layers with 256 and 128 filters, respectively. The RGCN had two graph convolution layers, and the filters were 256 and 128. The weighting factors, and were 0.2, 0.1, and 0.01, respectively. Our model's learning rate, weight decay and epoch count were set to 0.002, 0.0005 and 1065, respectively in the pan-cancer dataset and were fixed to 0.002, 0.0005 and 1000, respectively in the individual cancer datasets. The learning rate, weight decay and epoch count were set to 0.0001, 0.0005 and 1200, respectively for the baseline, multi-omics fusion method and were set to 0.0005, 0.0005 and 1500 for MOGONET, and 0.0005, 0 and 1000 for RGCN. MTGCN, EMOGI, GCN and GAT have the same number of convolution layers and the same filter size per layer. They had three graph convolution layers. The filters were set to 300, 100 and 1 for the pan-cancer dataset, and were set to 150, 50 and 1 for the sing cancer type dataset. Their learning rate is 0.001 and the epoch number is 2500. In predicting the two independent test sets, we adjusted our model's learning rate, weight decay and epoch number to 0.0005, 0.0005 and 1300, respectively for the OncoKB dataset and 0.00008, 0.0005, 700, respectively for the ONGene dataset. We set the weight decay of baselines to 0.005 according to the recommendation in [16].

### Experimental results

#### Prediction performance of pan-cancer driver genes

We applied our method and baselines to predict pan-cancer driver genes. Table 1 reports the average AUC and AUPRC values of each method under ten-fold cross-validation. Our MRNGCN controls the best performance in the AUC and AUPRC values. The AUC and AUPRC values of our model achieve 0.9192 and 0.8446, respectively, which are 0.72% and 1.14% higher than the second best method, MTGCN. The results demonstrate that multiple gene relationship networks help predict cancer driver genes. Although the baselines, like MOGNET, Multi-omics fusion and RGCN, also integrate the gene–gene, gene–outlying gene and gene–miRNA data to predict pan-caner driver genes, they even perform worse than MTGCN, which indicates cancer drivers based on the gene–gene network. The observed improvement in the performance of MRNGCN can be partially attributed to its successful integration of multi-omics data for cancer driver prediction.

**Table 1:** Performance comparison of pan-cancer driver genes prediction

Methods	AUC	AUPRC
MOGONET	0.8903 ± 0.0003	0.7922 ± 0.0010
Multi-omics fusion	0.9088 ± 0.0002	0.8246 ± 0.0007
RGCN	0.8973 ± 0.0002	0.8103 ± 0.0007
GCN	0.8855 ± 0.0002	0.7709 ± 0.0009
GAT	0.8576 ± 0.0003	0.6801 ± 0.0014
EMOGI	0.9044 ± 0.0003	0.8169 ± 0.0008
MTGCN	0.9116 ± 0.0002	0.8332 ± 0.0006
**MRNGCN**	**0.9192 ± 0.0002**	**0.8446 ± 0.0006**

Bold values indicates the best performance

AUC value and AUPRC comparison of our method MRNGCN and other comparison methods on pan-cancer dataset

#### Performance of cancer type-specific driver gene prediction

We also investigated the effectiveness of MRNGCN in detecting driver genes of a single cancer type, including breast cancer (BRCA), lung adenocarcinoma (LUAD), bladder cancer (BLCA) driver genes and hepatocellular carcinoma (LIHC). The positive samples of a single cancer type were from NCG6.0 labelled with that cancer type. There are 202, 179, 95, and 82 cancer driver genes in BRCA, LUAD, BLCA and LIHC, respectively. Their negative sample consisted of the same 2,187 genes as the pan-cancer data. For a single cancer type, we reorganized initial attributes for the nodes of the three networks. The initial gene attributes of a cancer type have 19 elements, consisting of three biological features of that cancer type and the structural features of length 16. The initial miRNA attributes consist of 4 elements, including the average miRNA expression value, average miRNA differential expression value, the number of connective genes and the miRNA-miRNA similarity feature of reduction to 1 dimension. The initial outlying gene attributes consist of an average z-score of the gene expression value and a frequency of the gene expression abnormal in the samples of the cancer type. We used the same method in subsection "Gene–outlying gene network" to get the outlying genes from the samples in every cancer type, involving 9691, 10,607, 9812 and 8593 outlying genes for LUAD, BRCA, BLCA and LIHC, respectively. Hence, our model constructed different gene–outlying gene networks for every cancer type and used the same gene–gene and gene–miRNA networks with the pan-cancer.

Table 2 reports the performance comparison between our model and baselines for single cancer-type driver gene prediction. As can be seen, MRNGCN has the highest AUC and AUPRC values compared to all baselines on the LUAD, BRCA, BLCA and LIHC datasets. For LUAD and BRCA with large positive sample sizes, our model's AUC and AUPRC values are 4% and 0.1%, 1% and 4% higher than the second best method MTGCN, respectively. For BLCA and LIHC with medium positive sample sizes, our model's AUC and AUPRC values are 1% and 7%, 3% and 8% higher than the second best method MTGCN, respectively. In Additional file 1: Table S1, we compared the performance of our model and other methods based on the 11 cancer type-specific driver gene predictions. We observed that our model outperforms baselines on most cancer-type datasets. It controls the highest AUPRC values on the 11 cancer type except in CESC and LUSC. As for the AUC value, our model performs better than baselines on 6 of 11 cancer-type datasets.

**Table 2:** Performance of cancer type-specific driver gene prediction

Types of cancer	AUC	AUPRC
*LUAD*		
MOGONET	0.8960 ± 0.0016	0.6106 ± 0.0074
Multi-omics fusion	0.8904 ± 0.0007	0.6221 ± 0.0039
RGCN	0.8720 ± 0.0017	0.4419 ± 0.0095
GCN	0.8042 ± 0.0014	0.4187 ± 0.0065
GAT	0.8180 ± 0.0018	0.3398 ± 0.0055
EMOGI	0.8709 ± 0.0019	0.5591 ± 0.0105
MTGCN	0.9019 ± 0.0012	0.6279 ± 0.0099
**MRNGCN**	**0.9427 ± 0.0011**	**0.6287 ± 0.0117**
*BRCA*		
MOGONET	0.8944 ± 0.0008	0.6350 ± 0.0032
Multi-omics fusion	0.9050 ± 0.0006	0.6714 ± 0.0030
RGCN	0.8557 ± 0.0029	0.3983 ± 0.0148
GCN	0.8813 ± 0.0009	0.5866 ± 0.0058
GAT	0.8673 ± 0.0044	0.4387 ± 0.0136
EMOGI	0.8989 ± 0.0007	0.6482 ± 0.0045
MTGCN	0.9061 ± 0.0006	0.6583 ± 0.0040
**MRNGCN**	**0.9120 ± 0.0008**	**0.6920 ± 0.0041**
*BLCA*		
MOGONET	0.9368 ± 0.0005	0.5884 ± 0.0111
Multi-omics fusion	0.9482 ± 0.0008	0.6539 ± 0.0153
RGCN	0.8420 ± 0.0023	0.4404 ± 0.0095
GCN	0.8712 ± 0.0013	0.3191 ± 0.0091
GAT	0.8929 ± 0.0007	0.2892 ± 0.0068
EMOGI	0.9359 ± 0.0005	0.5485 ± 0.0101
MTGCN	0.9495 ± 0.0005	0.6568 ± 0.0077
**MRNGCN**	**0.9544 ± 0.0005**	**0.7248 ± 0.0052**
*LIHC*		
MOGONET	0.8739 ± 0.0023	0.4306 ± 0.0198
Multi-omics fusion	0.8900 ± 0.0017	0.5220 ± 0.0082
RGCN	0.8648 ± 0.0024	0.5016 ± 0.0133
GCN	0.8098 ± 0.0019	0.3017 ± 0.0090
GAT	0.8472 ± 0.0014	0.2340 ± 0.0042
EMOGI	0.8753 ± 0.0023	0.3845 ± 0.0193
MTGCN	0.8937 ± 0.0016	0.4645 ± 0.0195
**MRNGCN**	**0.9109 ± 0.0012**	**0.5468 ± 0.0149**

Bold values indicates the best performance

AUC value and AUPRC comparison of our method MRNGCN and other comparison methods on specific cancer data sets, namely LUAD, BRCA, BLCA and LIHC

#### Performance of the independent test set

**Fig. 2:**
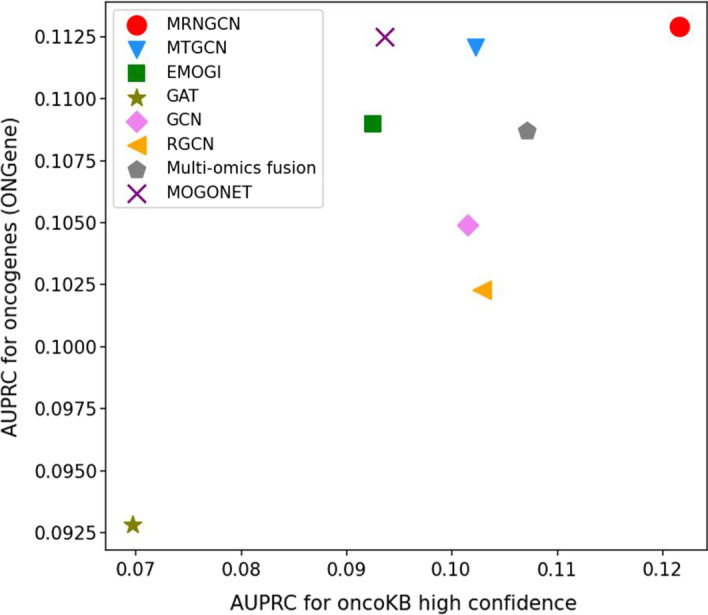
Performance comparison of different methods on two independent sets of cancer driver genes. The X-axis represents predicted AUPRC value of genes of oncoKB dataset and the Y-axis represents predicted AUPRC value of genes of ONGene dataset

We also compared the performance of MRNGCN and baselines on two independent datasets to investigate whether their performance is biased towards a particular dataset. We trained MRNGCN and baselines with all pan-cancer positive and negative samples, and then applied the trained models to predict genes in each independent test set. We defined the positive samples of the two independent test sets as those genes in the OncoKB [26] database or ONGene [27] database but not in the training set. After removing the training samples and the positive samples from the independent test set, the negative samples are all remaining genes. Figure 2 shows the AUPRC values for MRNGCN and baselines on the two independent test sets. The AUPRC values are low for all methods due to the more negative samples than the number of positive ones. However, we observed that MRNGCN performs better than baseline on both independent test sets.

#### Ablation experiments

The MRNGCN model learns gene features from the gene–gene, gene–outlying gene, and gene–miRNA networks conjunctively using some sub-modules, i.e., the HGCN models, self-attention layer, LR model, pre-training on the gene–miRNA network and the bilinear aggregation layer on the gene–gene network. We set up some model variants by inputting one or two networks or removing some sub-models to investigate which features or sub-modules are helpful for the MRNGCN model in predicting cancer driver genes. When removing the LR model, we applied a sigmoid function on the synthesis gene features($H\left( X \right)_{syn}$) to predict cancer driver genes. We also replaced the LR model with other popular classifiers, such as Random Forest and XGBoost, to verify the necessity of the LR model. The model did not consider the link prediction loss when we did not input the gene–gene network.

Table 3 demonstrates the performance comparison between the MRNGCN model and its variants in pan-cancer driver gene prediction. We observed that using one network performs worse than using two or three networks. The results of the three networks were the best (the AUC and AUPRC were 0.9192 and 0.8446), indicating that we successfully integrated the three networks for cancer driver identification. Especially the best results for a single network were achieved using the gene–miRNA network, whose AUC and AUPRC were 0.9128 and 0.8346, respectively. Moreover, combining the gene–miRNA network with the gene–gene (AUC = 0.9174 and AUPRC = 0.8428) or gene–outlying gene network (AUC = 0.9187 and AUPRC = 0.8440) performs better than integrating gene–gene or gene–outlying gene network (AUC = 0.9158 and AUPRC = 0.8407). It suggests the helpfulness of the gene–miRNA network in cancer driver predictions. We noticed removing the pre-training module decreased our model's prediction performance, which dropped 0.15% in the AUC value and 0.2% in the AUPRC value. If we applied a sigmoid function on the synthesis gene features to predict cancer driver genes instead of the LR model, the prediction performance drops 0.14% in the AUC value and 0.2% in the AUPRC value. If we replaced the LR model with random forest and XGBoost as the downstream classifier, the RF or XGBoost classifier models were 1.37% or 2.02% lower in AUC and 1.96% or 3.68% lower in AUPRC than our original model. These results suggest that the LR model may effectively combine different gene features to output the probability of a gene being a cancer driver. We also noticed that removing the bilinear aggregation layer when learning gene features from the gene–outlying gene network or removing the self-attention layer will slightly reduce the prediction performance (at most, reducing 0.07% in the AUC value and 0.08% in AUPRC value). Overall, the MRNGCN model had higher AUC and AUPRC values than all variants, suggesting that our model successfully improved the identification of cancer driver genes by integrating multiple gene relationship networks.

**Table 3:** Ablation experiments

Methods	AUC	AUPRC
Gene–gene network	0.9101 ± 0.0002	0.8312 ± 0.0006
Gene–outlying gene network	0.9093 ± 0.0002	0.8325 ± 0.0006
Gene–miRNA network	0.9128 ± 0.0003	0.8346 ± 0.0007
Gene–gene and gene–outlying gene networks	0.9158 ± 0.0002	0.8407 ± 0.0006
Gene–gene and gene–miRNA networks	0.9174 ± 0.0002	0.8428 ± 0.0006
Gene–outlying gene and gene–miRNA networks	0.9187 ± 0.0002	0.8440 ± 0.0006
No pre-training on the gene–miRNA network	0.9177 ± 0.0002	0.8426 ± 0.0007
Removal of bilinear aggregation layer	0.9185 ± 0.0002	0.8440 ± 0.0006
Removal of the self-attention layer	0.9180 ± 0.0002	0.8438 ± 0.0006
Removal of logistic regression model	0.9178 ± 0.0002	0.8426 ± 0.0006
Using Random Forest as classifier	0.9055 ± 0.0003	0.8250 ± 0.0007
Using XGBoost as classifier	0.8990 ± 0.0003	0.8078 ± 0.0010
**MRNGCN**	**0.9192 ± 0.0002**	**0.8446 ± 0.0006**

Bold values indicates the best performance

Comparison of AUC and AUPRC values of MRNGCN and its variants on pan-cancer dataset

#### Predicting new pan-cancer driver genes

To investigate the ability of MRNGCN to identify new pan-cancer driver genes, we trained our model with all positive and negative samples, and applied it to predict the unlabeled gene. Table 4 shows the top 30 candidate pan-cancer driver genes ranked by the MRNGCN, and their ranking positions in other methods (i.e. #MTGCN indicates the ranking position in MTGCN). We performed a co-citation analysis of these genes and listed the number of co-citations between genes and the keywords "cancer", "driver", "tumor" and "biomark", "drug target". We observed that all 30 genes are co-cited with the keyword "cancer" and 29 genes are associated with the keyword "driver". We also checked whether these genes were in the NCG 6.0 list and listed the tissues where the genes were located. 18 of 30 genes are recorded in the NCG database as driver genes of a cancer type. We also calculated the ratio of edges connected to known pan-cancer driver genes over all connective edges in the PPI network, and found that all genes except HCN1 connect to driver genes in the gene–gene network, consistent with the observation that driver genes tend to relate to each other performing functions. The Additional file 1 recorded the GO and pathway enrichment analysis for the 30 predicted cancer driver genes. All of these suggest that the top 30 genes can potentially be cancer-driver genes.

**Table 4:** Co-citer analysis of the top 30 genes predicted by MRNGCN

	#MRNGCN	#MTGCN	#EMOGI	#GAT	Cancer	Driver	Biomarker	Drug target	in_NCG	%interact with drivers (%)
VCAN	1	23	18	4092	28	1	1	0	Lung	18.18
NR3C1	2	10	14	673	65	4	4	1	Blood	25.91
EPHA2	3	19	23	582	113	2	4	4	pan-cancer_adult	31.82
LRP1	4	49	58	2544	54	3	5	1	Blood	14.13
STAT1	5	22	29	604	346	5	21	4		27.54
FOS	6	44	21	579	195	2	9	2		23.94
RASA1	7	42	24	163	20	1	0	1	head_and_neck, lung, stomach, multiple	45.05
HNF4A	8	27	25	305	37	1	5	2	Hepatobiliary	31.78
SP1	9	28	10	566	291	3	10	2		33.61
CCL11	10	90	106	8929	18	1	23	2		8.70
KLF5	11	80	64	345	64	1	3	0	Multiple, bladder, colorectal, bladder	42.31
CCL5	12	66	122	4948	111	1	18	0		13.95
LRP6	13	82	137	2194	65	1	2	3		13.33
ATF3	14	35	34	410	83	1	8	2		25.00
FLT1	15	152	105	78	162	1	57	3	Brain	30.30
HSPG2	16	43	55	998	12	3	5	2	Esophagus	20.51
RUNX2	17	68	97	390	93	3	9	2		37.70
IRAK1	18	39	104	1647	9	1	3	0		18.75
SHC1	19	11	4	365	122	4	9	2		26.79
EPHA4	20	337	169	633	11	1	0	1	Skin	30.30
TP53BP1	21	164	139	1269	76	1	2	0	Multiple	20.00
FN1	22	5	11	2971	76	1	16	0		12.11
APOB	23	29	39	4239	22	1	12	1	Multiple, hepatobiliary, brain, stomach	15.25
MACF1	24	25	32	3447	4	0	0	0	Pancreas, multi, stomach	18.33
HCN1	25	1265	388	9816	3	1	0	1	Lung	0.00
INHBA	26	101	80	2832	39	1	4	0	Lung	25.00
LYN	27	8	8	344	55	1	6	2	Blood	21.17
PLCG2	28	102	51	56	10	1	2	0	Multiple, blood	41.67
HOXA10	29	216	450	145	30	1	2	0	Multiple	20.00
NRP1	30	142	186	1223	55	1	1	1		16.95

The second to fifth columns of the table show the ranking of these thirty genes in other methods, and the sixth to ninth columns show the co- citation times of these genes and the keyword cancer, driver, biomarker and drug target, in_ NCG indicates whether these genes are in the NCG database. The last column indicates the proportion of the edges connected to these genes and the driver genes in all edges

**Fig. 3:**
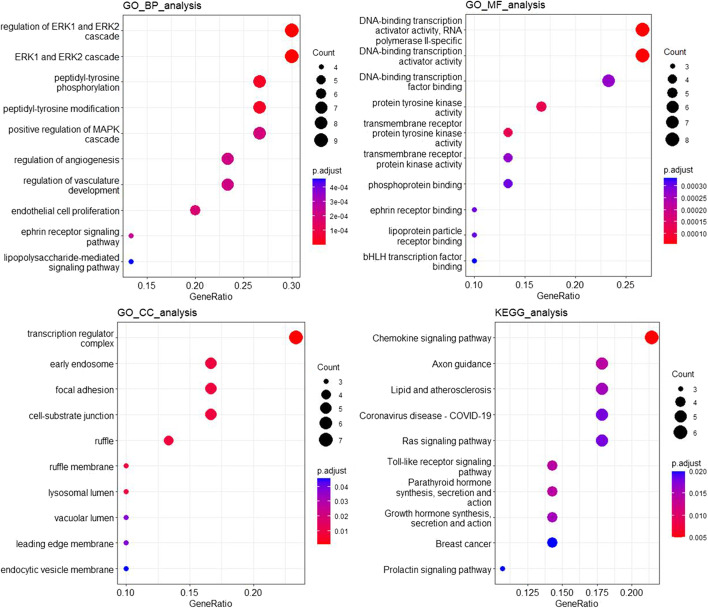
GO & pathway enrichment analysis for the 30 predicted cancer driver genes. This figure includes GO enrichment analysis from BP, CC and MF and KEGG enrichment analysis

Figure 3 illustrates the GO and pathway enrichment analysis for the 30 predicted cancer driver genes. In terms of biological process, the top 30 genes play more roles in regulation of EPR1 and ERK2 cascade, EPR1 and ERK2 cascade, peptidyl-tyrosine phosphorylation, peptidyl-tyrosine modification, positive regulation of MAPK cascade, regulation of angiogenesis, regulation of vasculature development, endothelial cell proliferation, ephrin receptor signaling pathway, lipopolysaccharide-mediated signaling pathway, etc. For cellular components, the 30 genes are considerably enriched in transcription regulator complex, early endosome, focal adhesion, cell-substrate junction, ruffle, ruffle membrane, lysosomal lumen, vacuolar lumen, leading-edge membrane and endocytic vesicle membrane, etc. For molecular functions, the 30 genes have the crucial functions of RNA polymerase II sequence-specific DNA binding transcription activator activity, DNA binding transcription activator activity, DNA binding transcription factor binding, protein tyrosine kinase activity, transmembrane receptor protein tyrosine kinase activity, transmembrane receptor protein kinase activity, phosphoprotein binding, ephrin receptor binding, lipoprotein particle receptor binding, bHLH transcription factor binding, etc. These 30 genes are enriched in the pathway of chemokine signaling pathway, Axon guidance, Lipid and atherosclerosis, coronavirus disease -covid-19, Ras signaling pathway, Toll-like receptor signaling pathway, parathyroid hormone synthesis, secretion and action, growth hormone synthesis, secretion and action, breast cancer, prolactin signaling pathway, etc. Thus, our model can find novel cancer driver genes for further experimental validation.

## Conclusion

In this study, we proposed a new approach called MRNGCN to identify cancer driver genes. It constructed three gene-related networks, including the gene–gene network, gene–outlying gene network and gene–miRNA network, and then ran three parameter–shared heterogeneous graph convolution network models on the three networks to learn node features. Moreover, we considered the relationship between genes with long distances and introduced the self-attentionon layer. The three gene features learned from the three networks are transformed to feature vectors of length 1 through the 1-dimensional convolutional modules and were fused by a 2-dimensional convolutional module. The cancer driver genes are predicted based on the probability scores of the combination of the gene features learned from the three networks, the fused gene features and the original gene features through an LR model. The model was optimized by minimizing the node and edge prediction loss. We implemented extensive experiments to test our model. The results show that: (1) Building multi-relational networks allows gene nodes to learn the features of neighbouring nodes, improving prediction performance. (2) MRNGCN performs significantly better than the baseline in a pan-cancer dataset, some single cancer type datasets and two independent test sets, demonstrating our approach's integration of multiple networks contributing to predicting cancer driver genes. (3) The ablation study showed that our model successfully improved the identification of cancer driver genes by integrating multiple gene relationship networks. Moreover, we observed that the gene–miRNA network helped to identify cancer driver genes.

Hence, MRNGCN is an effective feature-learning approach that employ multi-omics data to construct multiple gene relationship networks and integrate these networks to learn gene features for cancer driver identification. This model can be applied to other biological prediction problems, such as drug-drug association prediction [28] and PPI prediction [29]. We updated the node features by aggregating the features from its neighbors. However due to the modularity of the network, the nodes in the same modules may share features. In the future work, we consider using clustering methods [30–32] to partition the network and filter features to improve the model performance.

## Supplementary Information

**Additional file 1.** Supplementary material.

## Data Availability

The Somatic mutations, miRNA and mRNA expression data were downloaded from TCGA (https://portal.gdc.cancer.gov/). All codes necessary to re-generate the results are publicly available at https://github.com/weiba/MRNGCN.
