# Supplementary material for: Identifying cancer driver genes based on multi-view heterogeneous graph convolutional network and self-attention mechanism
Source: BMC Bioinformatics. 2023 Jan 13;24:16. doi: 10.1186/s12859-023-05140-3 (PMC9838012; doi:10.1186/s12859-023-05140-3)
Supplement: Supplementary file 1 — Additional file 1. Supplementary material. [file 12859_2023_5140_MOESM1_ESM.pdf]

Supplementary file of " Identifying cancer driver genes based on multi-view heterogeneous graph convolutional network and self-attention mechanism"

1.Building multi-relational networks

1.1 Gene-gene network

We used the PPI network to construct the gene-gene network. Let $A_{PP} \in \{0,1\}^{n\times n}$ be the adjacency matrix of the gene-gene network with the number n of genes. If two genes connect through an edge in the PPI network, the corresponding value in the matrix A_{PP} is 1. Otherwise, it is 0.

We took the same way of MTGCN[1] to prepare initial attributes for gene nodes in the networks. Let $X_P \in R^{n\times F_1}$ denote the initial gene attributes consisting of biological and topological features. For each cancer type, we calculated gene mutation rate, gene differential DNA methylation rate, and gene differential expression rate as the biological features of the genes. The gene mutation rate was defined as the average times of the gene containing single nucleotide variants (SNVs) or copy number aberrations (CNAs) across all samples in that cancer type. The differential DNA methylation rate for each gene is the average of the differences in methylation signals between cancer and matched normal samples across all samples of the cancer type, defined as follows.

$$dm_i^c = \frac{1}{|S_c|}\sum_{s\in S_c}(\beta_i^t - \beta_i^n) \quad (S1)$$

where dm_i^c represents the DNA methylation value of the gene i in the cancer type c. β_i^t and β_i^n are methylation signals in cancer and matched normal samples. S_c denotes the set of cancer samples. The differential expression rate of a gene was calculated by the log2 fold change between the expression value of the cancer sample and the matched normal sample in each cancer and then averaged across all samples. Since we only focused on 16 cancer types, each gene had a 48-dimensional biological feature vector, including 16 mutation rates, 16 methylation values, and 16 differential expression rates, which were min-max normalized. The 16-dimensional topological features of genes resulted from the note2Vec[2] on the gene-gene network. We concatenated the 48-dimensional biological features and the 16-dimensional topological features to get the 64-dimensional initial attributes of genes.

1.2 Gene-outlying gene network

We considered that a driver gene usually affects the expression of genes linked to it in a biological network, so we constructed a gene-outlying gene network. A gene is considered to be outlying if the absolute of its z-score is above 2 . The z-score of a gene in a sample is defined as follows.

$$z - score = \frac{x - \mu}{\sigma} \quad (S2)$$

Where x is the gene expression value in a sample, μ and σ represent the mean and variance of the expression values of genes in a sample. We collected all outlying genes that were at least expressed abnormally in one sample of the 16 cancer types. Let $A_{PO} \in \{0,1\}^{n\times m}$ be the adjacency matrix of the gene-outlying network with the number n of genes and m of outlying genes. We connected a gene and an outlying gene and set the corresponding value of A_{PO} as 1 if the gene mutated in at least one cancer sample and there was an edge linking it and the outlying gene in the PPI network. Hence, the gene-outlying gene network contains 13,627 gene nodes, 12,248 outlying gene nodes and 469,078 edges.

We initialized the attributes of the gene nodes in the gene-outlying network as X_P, the same attributes of the gene nodes in the gene-gene network. The initial attributes of the outlying genes consist of the average z-scores across all samples of a cancer type and the frequencies of being outlying among the samples of the cancer type. Let $X_O \in R^{m\times F_2}$ be the vector of the initial attributes of the outlying genes. For the pan-cancer with 16 cancer types, X_O would consist of 16-dimensional z-score features and 16-dimensional frequency features. For convenience, the outlying gene initial features underwent linearly transformation from 32 dimensions to 64, the dimension of initial gene features.

1.3 Gene - miRNA network

We constructed a gene-miRNA network considering the regulatory relationship of miRNAs on gene expression. The known associations between miRNAs and their targeted genes were from mirTarbase V8.0[3]. Let $A_{PR} \in \{0,1\}^{n\times t}$ be the adjacency matrix of the gene-miRNA network with n genes and t miRNAs. The genes were the mutated gene of TGCA samples. The miRNAs were those that both appear in the mirTarbase V8.0 database and the TGCA samples. The value of A_{PR} is 1 if a gene is associated with a miRNA. Otherwise, it is 0. Hence, the gene-miRNA network contains 13,627 gene nodes, 1,390 miRNA nodes and 153,913 edges.

We also initialized the attributes of the gene nodes in the gene-miRNA network as X_P, the same attributes of the gene nodes in the gene-gene network. The initial attributes of miRNAs denoted by $X_R \in R^{t\times F_3}$, including the average z-scores and the average different expression values across all samples of every cancer type and the similarities with other miRNAs. We calculated the differential expression values of miRNAs in each cancer sample as follows.

$$\begin{cases} log_2FC(A,B) = log_2(\frac{B}{A}) \\ Delta(\delta) = log_2FC \times (B - A) \end{cases} \quad (S3)$$

where A and B represent the miRNA expression values of normal and cancer samples, respectively. Since miRNAs link to the pathologies of cancers by regulating the expression of their targeted genes and the dysfunction of similar miRNAs would lead to a similar phenotype, we introduced the miRNA similarities as part of initial miRNA attributes. Similar to previous works[4], the miRNA similarity was measured by the miRNA GIP (Gaussian Interaction Profile) kernel based on known miRNA-disease associations. Let $IP(m_i)$ represent the known interaction profile of miRNA m_i with diseases. For two miRNAs, m_i and m_j, their GIP similarity is defined in Equation (4).

$$KM_{GIP}(m_i, m_j) = exp(-\gamma_m \|IP(m_i) - IP(m_j)\|^2) \quad (S4)$$

$$\gamma_m = \gamma'_m \Bigg/ \left(\frac{1}{n}\sum_{i=1}^{t} \|IP(m_i)\|^2\right)$$

Where γ'_m is set to 1. t is the number of miRNAs. We passed the miRNA GIP simiarity matrix through a linear layer and obtained 16-dimensional miRNA similarity features to avoid bias. Finally, for the pan-cancer with 16 cancer types, X_R would have 16-dimensional z-scores and 16-dimensional differential expression values and 16-dimensional miRNA similarities. Similarly, for convenience, we linearly transformed the miRNA initial features to the same dimension as initial gene features. In this work, we introduced gene-miRNA associations for cancer driver prediction. In the following steps, I will update gene features using the features of its connective miRNAs. The whole model is optimized by minimizing loss between the known node labels and the predicted ones. However, the number of node labels is limited. To fully use the valuable gene-miRNA associations, we implemented pre-training on the gene-miRNA network to learn new features for genes and miRNA, called $X_{P_{pre}}$ and $X_{R_{pre}}$. I leveraged a two-layer heterogeneous graph convlutional network (HGCN) model the same as the model in section 3.3.1 to pre-train and learn $X_{P_{pre}}$ and $X_{R_{pre}}$. Let $P_{PR} = D^{-\frac{1}{2}}AD^{\frac{1}{2}}$ be the normalized matrix of the adjacent matrix A_{PR}. $D_P = \sum_R A_{PR} + 1$ and $D_R = \sum_R A_{RP} + 1$. $A_{PR} = A_{RP}^T$, $P_{PR} = P_{RP}^T$. σ is the activation function. The $X_{P_{pre}}$ and $X_{R_{pre}}$ can be calculated by following equations.

$$X^1_{P_{pre}} = \sigma(P_{PR}X_R\theta^1_t + P_{PR}X_R \odot X_P W^1_t + b^1_t) \quad (S5)$$

$$X^1_{R_{pre}} = \sigma(P_{RP}X_P\theta^1_t + P_{RP}X_P \odot X_R W^1_t + b^1_t) \quad (S6)$$

$$X_{P_{pre}} = X_P + \sigma(P_{PR}X^1_{R_{pre}}\theta^2_t + P_{PR}X^1_{R_{pre}} \odot X^1_{P_{pre}} W^2_t + b^2_t) \quad (S7)$$

$$X_{R_{pre}} = X_R + \sigma(P_{RP}X^1_{P_{pre}}\theta^2_t + P_{RP}X^1_{P_{pre}} \odot X^1_{R_{pre}} W^2_t + b^2_t) \quad (S8)$$

Here, θ^1_t, W^1_t, b^1_t, θ^2_t, W^2_t, b^2_t are learnable parameters. We calculated the inner product of $X_{P_{pre}}$ and $X_{R_{pre}}$ to obtain the reconstructed adjacency matrix $\widehat{A}_{PR}$. The pre-training model was optimized by minimizing the difference between the matrix $\widehat{A}_{PR}$ and A_{PR}. For the loss function, please see Equation(S10). Hence, we used $X_{P_{pre}}$ and $X_{R_{pre}}$ as the gene and miRNA initial features of the gene-miRNA network for following feature learning. σ is the sigmoid function

$$\widehat{A}_{PR} = \sigma(X_{P_{pre}}\ X_{R_{pre}}) \quad (S9)$$

$$L_{r\_loss} = -\frac{1}{n}\left\{\sum_{(i,j)\in E}\{log\hat{a}_{i,j}\} + \sum_{(i,j)\in Neg}\{1 - log\hat{a}_{i,j}\}\right\} \quad (S10)$$

Where E is the edge set of the gene-gene network, and n is the size of E. Neg is the set of negative samples with size n, obtained by negative sampling, and $\hat{a}_{i,j}$ is the value of the reconstructed adjacency matrix.

2. The structure of two-dimensional convolutional layer

The 2D-convolution module consists of a two-dimensional convolutional layer (see Figure S1 for detailed structures). Firstly, we stack the three gene features from the output of the 1D-convolution module to obtain a feature matrix $H(X)_{stack} \in R^{3\times n\times 1}$, with n denoting the number of gene nodes. Then we padded out a circle of zeros around the $H(X)_{stack}$ and implemented two-dimensional convolutional operations on the $H(X)_{stack}$. The convolution kernel size is $(w_c \times h_c)$ and the perceptual field of $H(X)_{stack}$ is $3 \times w_c \times h_c$. Here, we set $w_c = h_c = 3$. The input channel is 3, and the output channel is 1. Hence, for the elements of $H(X)_{stack}$ on each channel at the ith row and jth column, the feature map can be expressed as follows.

$$H(X)_{stack}(i,j) = H(X)_{stack}[1{:}n_{in}, i{:}i + w_c, j{:}j + h_c]$$
$$Y(i,j) = H(X)_{stack}(i,j)W_3 + b_3 \quad (S11)$$

Where $1 \le i \le 2 + \mathrm{n} - w_c$, $1 \le j \le 3 - h_c + 1$, W_3 and b_3 denote the learnable parameters, respectively. We summed the feature maps on each channel to form the fused gene features learned by the 2D-convolution module, $H(X)_{2D} \in R^{n\times 1}$.

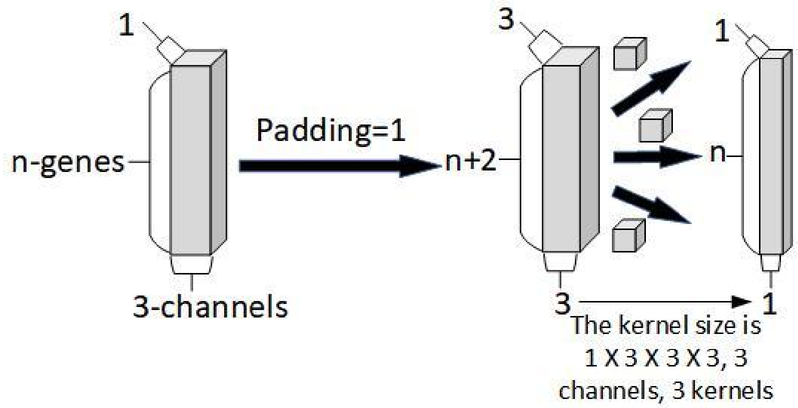

Figure S1. two-dimensional convolutional layer

3. The pseudo-code for MRNGCN

Algorithm 1: **MRNGCN** method

Input: Data source mentioned in the Data materials section, gene labels $\boldsymbol{y}$, number of HGCN layers $\boldsymbol{L}$, HGCN filters $\boldsymbol{a^i}, i = 1, \ldots, L$, dropout rate $\boldsymbol{d}$, weighting coefficients $\boldsymbol{\alpha}$, $\boldsymbol{\omega_1}$ and $\boldsymbol{\omega_2}$, epoch number $\boldsymbol{e}$, initial learning rate $\boldsymbol{lr}$, weight decay $\boldsymbol{w}$

Output: predicted value of each gene.

1: Building gene-gene network $\boldsymbol{G_{PP}}$ adjacency matrix $\boldsymbol{A_{PP}}$ and initial gene attributes $\boldsymbol{X_P}$

2: Building gene-outlying gene network $\boldsymbol{G_{PO}}$ adjacency matrix $\boldsymbol{A_{PO}}$ and initial outlying gene attributes $\boldsymbol{X_O}$

3: Building gene-miRNA network $\boldsymbol{G_{PR}}$ adjacency matrix $\boldsymbol{A_{PR}}$ initial miRNA attributes $\boldsymbol{X_R}$. Implement

pre-train on the gene-miRNA network to obtain gene features $X_{P_{pre}}$, miRNA features $X_{R_{pre}}$

4：Training.

for i = 1 to E do.

$\boldsymbol{P_{PP}}$, $\boldsymbol{P_{PO}}$ and $\boldsymbol{P_{PR}}$ are normalized adjacency matrixes of $\boldsymbol{A_{PP}}$, $\boldsymbol{A_{PO}}$, $\boldsymbol{A_{PR}}$, respectively

Calculating $\boldsymbol{H(X)_{P1}, H(X)_{P2}, H(X)_{P3}}$ by ***Equation(8)***

Calculating $\boldsymbol{H(X)_{P}^{BA}}$ by ***Equation (9)***

$\widetilde{H}(X)_P^2 = (1-\alpha) * H(X)_P^2 + \alpha * H(X)_P^{BA})$

Calculating $\boldsymbol{H(X)_{att}^1, H(X)_{att}^2, H(X)_{att}^3}$ by ***Equation(14)***

$\boldsymbol{H(X)_{1D}^i} = 1DconvModule(\boldsymbol{H(X)_{att}^i}), i = 1,2,3$

$\boldsymbol{H(X)_{2D}} = 2DconvModule(\boldsymbol{H(X)_{stack}})$

of which

$\boldsymbol{H(X)_{stack}} = pile\ up\{\boldsymbol{H(X)_{1D}^i}\}$

$H(X)_{mlp} = Linear^3(\sigma(Linear^2(\sigma(Linear^1(X_P)))))$

$\boldsymbol{H(X)_{ysn} = H(X)_{2D} + H(X)_{mlp}}$

Node prediction task loss:

$\boldsymbol{\hat{y}} = \sigma(\boldsymbol{H(X)_{syn}})$,$\boldsymbol{\hat{y}_1} = \sigma(\boldsymbol{H(X)_{2D}})$,

calculating $\boldsymbol{L_{n\_loss}}$,$\boldsymbol{L_{n\_loss1}}$ by ***Equations (15)*** and ***(16)***

Link prediction task loss:

Reconstruct adjacency matrix $\boldsymbol{\hat{A}_{PP}}$.

$\boldsymbol{\hat{A}_{PP}} = \sigma(\{\boldsymbol{H(X)_{1D}^1}\}\{\boldsymbol{H(X)_{1D}^1}^T\})$

calculating $\boldsymbol{L_{r\_loss}}$ by *Equation(18)*

$\boldsymbol{L_{total}} = \boldsymbol{L_{n\_loss}} + \omega_1 * \boldsymbol{L_{n\_loss1}} + \omega_2 * \boldsymbol{L_{r\_loss}}$

By minimizing $\boldsymbol{L_{total}}$ to optimize model parameters

end for.

5: test

Obtaining the predicted scores of the test genes by LR model

4. The framework of Multi-omics fusion

Multi-omics fusion concatenates the gene features learned from the three networks (i.e., $H(X)_{att}^1$, $H(X)_{att}^2$, $H(X)_{att}^3$) and the original gene features, X_P and then passes them through three fully connected layers with 256, 64, and 1 units to acquire gene fused features. It utilizes the fused features to finish the node prediction task and uses the gene features from the gene-gene network, $H(X)_{att}^1$ to do the link predict task.

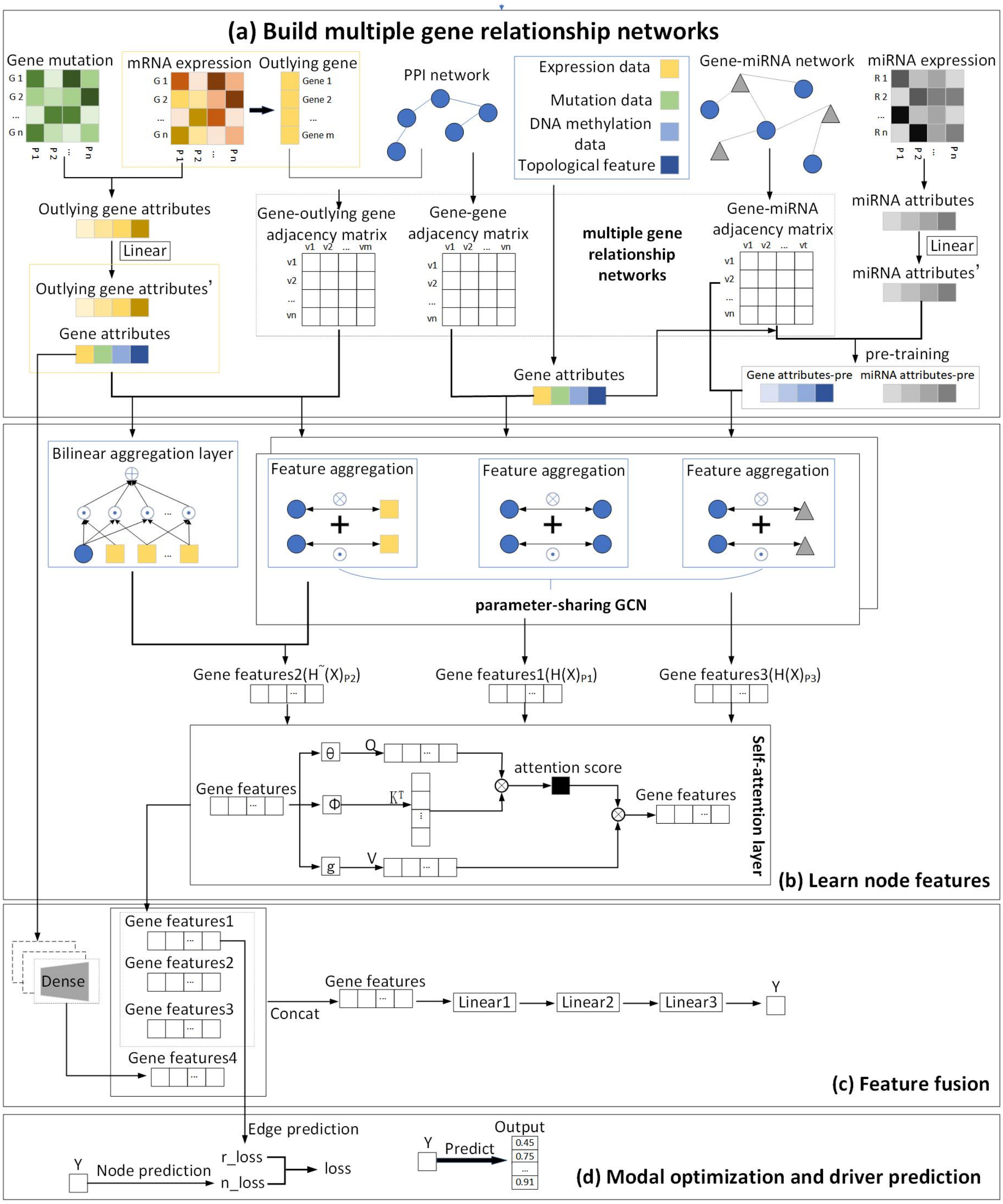

Figure S2. The framework of Multi-omics fusion

5. The framework of MOGONET

MOGONET was originally proposed to integrate multi-omics data to classify cancer samples. To evaluate the effectiveness of our feature fusion strategies, we input the gene features learned from the three networks by our model to the feature fusion module of MOGONET to predict cancer driver genes.

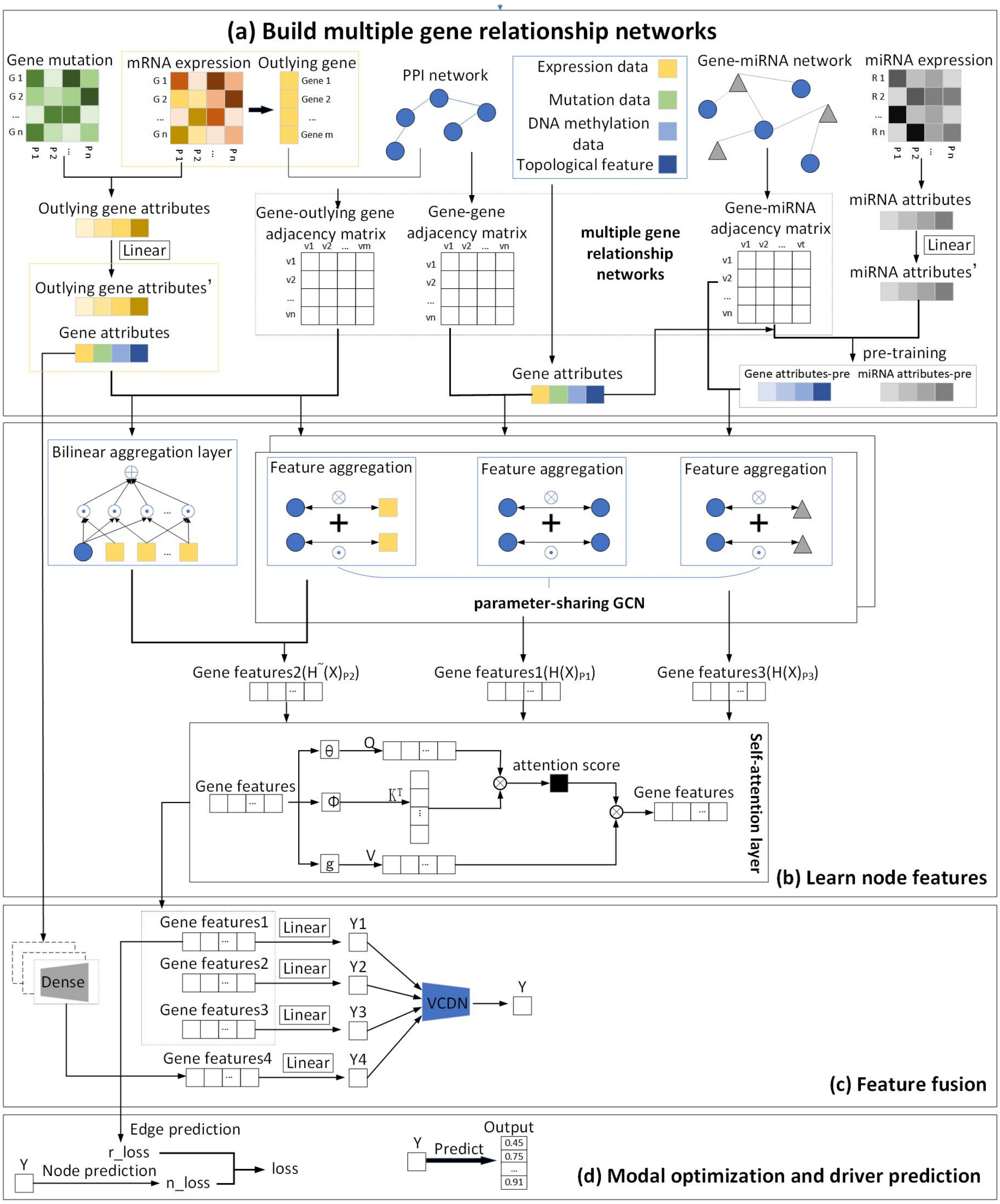

Figure S3 The framework of MOGONET

6. Performance of single cancer type-driven gene prediction

Since the NCG6.0 database does not record driver genes for rectal adenocarcinoma (READ) , we compared the performance of our model and other methods based on the rest 11 cancer type-specific driver gene predictions. We observed that our model outperforms baselines on most cancer-type datasets. It controls the highest AUPRC values on the 11 cancer type except in CESC and LUSC. As for the AUC value, our model performs better than baselines on 6 of 11 cancer-type datasets.

Table S1. Performance comparison on 11 cancer types dataset

Types of cancer		AUC	AUPRC
CESC	MOGONET	0.9671±0.0017	0.5448±0.0751
	Multi-omics fusion	0.9614±0.0044	0.5510±0.0736
	RGCN	0.8309±0.0036	0.3633±0.0108
	GCN	0.8201±0.0133	0.0980±0.0157
	GAT	0.9001±0.0039	0.0839±0.0089
	EMOGI	0.9740±0.0008	0.5547±0.0522
	**MTGCN**	**0.9805±0.0005**	**0.5972±0.0528**
	MRNGCN	0.9627±0.0033	0.5339±0.0694
COAD	MOGONET	0.8048±0.0017	0.3392±0.0050
	Multi-omics fusion	0.8165±0.0013	0.3864±0.0058
	RGCN	0.8374±0.0020	0.3875±0.0119
	GCN	0.7630±0.0021	0.2680±0.0048
	GAT	0.7828±0.0011	0.1932±0.0015
	EMOGI	0.8060±0.0011	0.3459±0.0067
	MTGCN	0.8295±0.0013	0.3816±0.0085
	**MRNGCN**	**0.8502±0.0014**	**0.4953±0.0067**
ESCA	MOGONET	0.8778±0.0021	0.4197±0.0127
	Multi-omics fusion	0.8947±0.0020	0.4778±0.0182
	RGCN	0.8361±0.0026	0.3555±0.0177
	GCN	0.8129±0.0027	0.2959±0.0106
	GAT	0.8530±0.0019	0.2478±0.0118
	EMOGI	0.8673±0.0040	0.4174±0.0297
	MTGCN	0.9020±0.0024	0.4721±0.0279
	**MRNGCN**	**0.9187±0.0008**	**0.5366±0.0121**
HNSC	MOGONET	0.8077±0.0021	0.3796±0.0077
	Multi-omics fusion	0.8161±0.0015	0.4378±0.0080
	RGCN	0.8525±0.0024	0.4098±0.0093
	GCN	0.7831±0.0018	0.3290±0.0051
	GAT	0.7881±0.0040	0.2426±0.0047
	EMOGI	0.8131±0.0019	0.3999±0.0078
	MTGCN	0.8387±0.0008	0.4396±0.0060
	**MRNGCN**	**0.8695±0.0011**	**0.5442±0.0062**
KIRC	MOGONET	0.8796±0.0042	0.3726±0.0226
	Multi-omics fusion	0.9023±0.0035	0.4265±0.0328
	RGCN	0.8224±0.0029	0.3328±0.0122
	GCN	0.7669±0.0066	0.0861±0.0057
	GAT	0.8351±0.0029	0.0906±0.0042
	EMOGI	0.8992±0.0027	0.3438±0.0235
	MTGCN	**0.9078±0.0024**	0.3498±0.0249
	MRNGCN	0.9032±0.0035	**0.4097±0.0240**
	MOGONET	0.9557±0.0009	0.3527±0.0384

KIRP	Multi-omics fusion	0.9155±0.0093	0.3823±0.0442
	RGCN	0.8414±0.0043	0.5089±0.0128
	GCN	0.7012±0.0091	0.0235±0.0002
	GAT	0.8271±0.0062	0.0693±0.0051
	EMOGI	0.9351±0.0016	0.2449±0.0289
	MTGCN	0.9645±0.0006	0.4358±0.0388
	**MRNGCN**	**0.9688±0.0008**	**0.5967±0.0428**
LUSC	MOGONET	0.8654±0.0074	0.2303±0.0267
	Multi-omics fusion	0.8064±0.0174	0.2574±0.0361
	RGCN	0.8113±0.0031	**0.3909±0.0102**
	GCN	0.7187±0.0145	0.0401±0.0007
	GAT	0.8131±0.0067	0.0673±0.0021
	EMOGI	0.8411±0.0091	0.2222±0.0266
	MTGCN	0.8984±0.0062	0.3099±0.0407
	MRNGCN	**0.9273±0.0028**	0.3478±0.0316
PRAD	MOGONET	0.9117±0.0029	0.5134±0.0205
	Multi-omics fusion	0.8963±0.0046	0.5674±0.0264
	RGCN	0.8164±0.0033	0.3758±0.0134
	GCN	0.8304±0.0050	0.3010±0.0185
	GAT	0.8475±0.0133	0.1777±0.0097
	EMOGI	0.9094±0.0025	0.5233±0.0220
	MTGCN	0.9170±0.0024	0.6143±0.0189
	**MRNGCN**	**0.9282±0.0028**	**0.6688±0.0164**
STAD	MOGONET	0.8795±0.0033	0.4975±0.0214
	Multi-omics fusion	0.8880±0.0026	0.5172±0.0136
	RGCN	0.8611±0.0030	0.4065±0.0136
	GCN	0.8176±0.0025	0.2502±0.0090
	GAT	0.8260±0.0018	0.1717±0.0039
	EMOGI	0.8883±0.0015	0.4604±0.0153
	MTGCN	**0.9265±0.0012**	0.5931±0.0117
	MRNGCN	0.9222±0.0014	**0.6458±0.0111**
THCA	MOGONET	0.8337±0.0029	0.2851±0.0134
	Multi-omics fusion	0.8279±0.0030	0.2812±0.0116
	RGCN	0.8215±0.0026	0.3067±0.0119
	GCN	0.7725±0.0040	0.1519±0.0077
	GAT	0.7966±0.0023	0.0976±0.0009
	EMOGI	0.7975±0.0020	0.2587±0.0084
	MTGCN	0.8263±0.0017	0.2907±0.0110
	**MRNGCN**	**0.8347±0.0044**	**0.3080±0.0102**
UCEC	MOGONET	0.8630±0.0034	0.3834±0.0174
	Multi-omics fusion	0.8802±0.0018	0.4302±0.0132
	RGCN	0.8339±0.0022	0.3782±0.0126
	GCN	0.8416±0.0026	0.2550±0.0133

	GAT	0.8447±0.0016	0.1568±0.0031
	EMOGI	0.8713±0.0017	0.4002±0.0143
	MTGCN	**0.9066±0.0014**	0.4954±0.0145
	MRNGCN	0.9061±0.0026	**0.5697±0.0148**

7. The coefficients in the LR model interpret every part's contribution

We took all positive and negative pan-cancer samples to train our model. The LR model combines the gene features from the three networks, the fused gene features and the original gene features that undergo linear transformation to determine whether the gene is a cancer driver. We got the coefficients of the LR model and implemented the maximum- minimum normalization on them. The coefficients X1, X2, and X3 were related to the gene features learned from the three networks. Y1 and Y2 were related to the fused and original gene features. Figure S4 reflect the contribution of each part to the driver gene identification. As we can see, the original gene features Y2 play a crucial role in driver gene identification. The second important feature is the fused features Y1 that integrate the features learned from the three gene relationship networks.

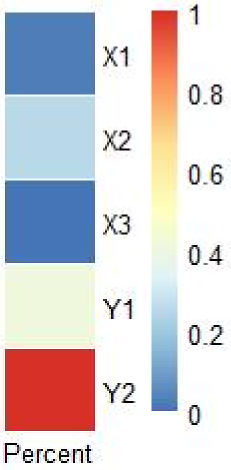

Figure S4. the importance of X1, X2, X3, Y1, Y2

8. Predicting new pan-cancer driver genes

To investigate the ability of MRNGCN to identify new pan-cancer driver genes, we trained our model with all positive and negative samples and applied it to predict the unlabeled gene. Table 4 shows the top 30 candidate pan-cancer driver genes ranked by the MRNGCN and their ranking positions in other methods (i.e. #MTGCN indicates the ranking position in MTGCN). We performed an online co-citation analysis[5] of these genes and listed the number of co-citations between genes and the keywords "cancer", "driver", "tumor" and "biomark", "drug target". We observed that all top 30 genes are co-cited with the keyword "cancer" and 29 genes are associated with the keyword "driver". The higher the number, the more likely the gene is to be cited and the more likely it is to be a cancer-driver gene. We also checked whether these genes were in the NCG 6.0 list and listed the tissues where the genes were located. 18 of 30 genes are recorded in the NCG database as driver genes of a cancer type. We also calculated the ratio of edges connected to

known pan-cancer driver genes over all connective edges in the gene-gene network and found that all genes except HCN1 connect to driver genes in the gene-gene network, which is consistent with the observation that driver genes tend to connect together to perform functions.

Figure S5 illustrates the GO and pathway enrichment analysis for the 30 predicted cancer driver genes. In terms of biological process, the top 30 genes play more roles in regulation of EPR1 and ERK2 cascade, EPR1 and ERK2 cascade, peptidyl-tyrosine phosphorylation, peptidyl-tyrosine modification, positive regulation of MAPK cascade, regulation of angiogenesis, regulation of vasculature development, endothelial cell proliferation, ephrin receptor signaling pathway, lipopolysaccharide-mediated signaling pathway, etc. For cellular components, the 30 genes are considerably enriched in transcription regulator complex, early endosome, focal adhesion, cell-substrate junction, ruffle, ruffle membrane, lysosomal lumen, vacuolar lumen, leading-edge membrane and endocytic vesicle membrane, etc. For molecular functions, the 30 genes have the crucial functions of RNA polymerase II sequence-specific DNA binding transcription activator activity, DNA binding transcription activator activity, DNA binding transcription factor binding, protein tyrosine kinase activity, transmembrane receptor protein tyrosine kinase activity, transmembrane receptor protein kinase activity, phosphoprotein binding, ephrin receptor binding, lipoprotein particle receptor binding, bHLH transcription factor binding, etc. These 30 genes are enriched in the pathway of chemokine signaling pathway, Axon guidance, Lipid and atherosclerosis, coronavirus disease -covid-19, Ras signaling pathway, Toll-like receptor signaling pathway, parathyroid hormone synthesis, secretion and action, growth hormone synthesis, secretion and action, breast cancer, prolactin signaling pathway, etc. Thus, our model can find novel cancer driver genes for further experimental validation.

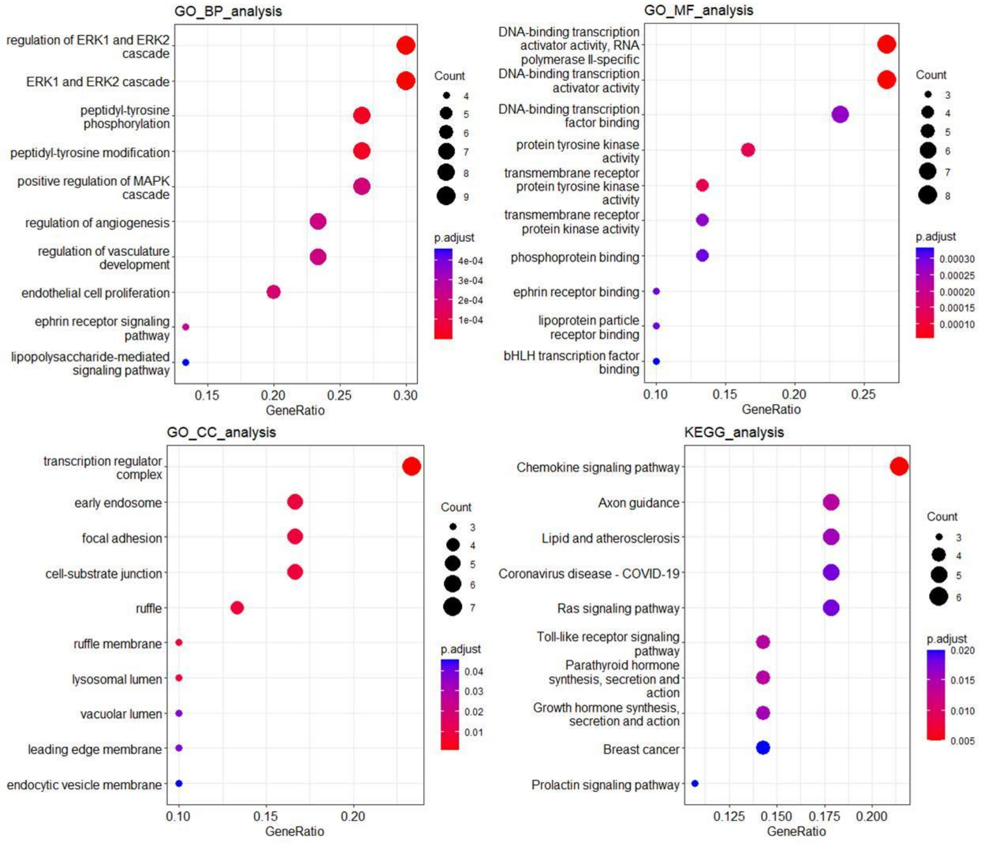

Figure S5 GO & pathway enrichment analysis for the 30 predicted cancer driver genes.

Reference

[1] Wei Peng, Qi Tang, Wei Dai, Tielin Chen, Improving cancer driver gene identification using multi-task learning on graph convolutional network, Briefings in Bioinformatics, 2021;, bbab432

[2] Grover, A. and J. Leskovec, *node2vec: Scalable Feature Learning for Networks.* KDD, 2016. **2016**: p. 855-864.

[3] H. Dweep, and N. Gretz, "miRWalk2. 0: a comprehensive atlas of microRNA-target interactions," Nature methods, vol. 12, no. 8, pp. 697-697, 2015.

[4] Peng W , Du J , Dai W , et al. Predicting miRNA-Disease Association Based on Modularity Preserving Heterogeneous Network Embedding[J]. Frontiers in Cell and Developmental Biology, 2021, 9:603758.

[5] Qiao, N., et al., CoCiter: An Efficient Tool to Infer Gene Function by Assessing the Significance of Literature Co-Citation. PLOS ONE, 2013. 8(9): p. 74074. e74074.
